# Comparative study of brain activity and functional connectivity in blepharospasm and blepharospasm-oromandibular dystonia

**DOI:** 10.3389/fneur.2025.1583297

**Published:** 2025-07-03

**Authors:** Yanying Wang, Min Luo, Bo Hou, Yang Li, Yingmai Yang, Feng Feng, Lin Wang, Xinhua Wan

**Affiliations:** ^1^Department of Neurology, Peking Union Medical College Hospital, Chinese Academy of Medical Sciences, Peking Union Medical College, Beijing, China; ^2^Department of Radiology, Peking Union Medical College Hospital, Chinese Academy of Medical Sciences, Peking Union Medical College, Beijing, China

**Keywords:** blepharospasm, blepharospasm-oromandibular dystonia, dynamic ALFF, dynamic FC, fMRI

## Abstract

**Background:**

The most common spread of blepharospasm (BSP) is to the oromandibular region, labeled as blepharospasm-oromandibular dystonia (BOM). We aimed to identify shared and different functional changes in BSP and BOM, trying to unveil the pathogenesis of these disorders and the mechanism of dystonic spread.

**Materials and methods:**

This single center study recruited 16 BSP patients, 16 BOM patients and 20 healthy controls (HC). Clinical information and resting-state fMRI images were collected. Dynamic amplitude of low-frequency fluctuations (dALFF) was calculated using the sliding window method. Intergroup differences in static ALFF (sALFF) and dALFF were examined. Using dALFF results, seed-based static and dynamic functional connectivity (FC) were constructed to compare connectivity changes in BSP and BOM networks. Correlations between dynamic parameters and disease severity scores were analyzed using Spearman partial correlation.

**Results:**

Compared with HC, BSP and BOM presented increased dALFF in the bilateral basal ganglia, bilateral supplementary motor area, right precentral gyrus, and bilateral cingulate gyrus. BOM further demonstrated decreased sALFF in the left cerebellum. Compared with HC, BOM patients had decreased sFC in the network involving the sensorimotor cortex, supplementary motor area, basal ganglia, cerebellum, and brainstem. In addition, decreased dFC strength was found between the right pallidum and cerebellum. Comparing with BSP patients, BOM patients showed decreased sFC and dFC strength in a similar but limited pattern. Clinical scores of BSP severity were significantly correlated with dALFF in some of these important regions.

**Conclusions:**

Our results demonstrated common brain regions with impaired functional activity in BSP and BOM patients. Further, BOM is featured with widespread connectivity reduction in the sensorimotor cortico-basal ganglia-brainstem-cerebellar network deriving from these key regions. These findings could help investigate mechanisms of dystonia spread and potentially facilitate disease-modifying therapies.

## Introduction

Blepharospasm (BSP) is a focal dystonia characterized by involuntary spasms of the orbicularis oculi muscle leading to eyelid closure (1). Compared with other focal dystonia, BSP has a higher risk and faster speed of dystonic spread (2, 3). The most common spread is to the oromandibular region, manifesting as jaw clenching, jaw opening, jaw deviation, and tongue protrusion (2, 4). This disease is labeled blepharospasm-oromandibular dystonia (BOM), also known as Meige syndrome. BSP affects facial expressions and lead to functional blindness, while BOM additionally results in eating and speech difficulties, severely impacting quality of life. Neuroelectrophysiology studies of BSP and BOM patients revealed reduced inhibition of the motor system, including the brainstem, basal ganglia, and cortical inhibitory regions (5). Moreover, BOM tends to have more extensive cortical inhibition impairments than does BSP (6). However, the specific etiology and pathophysiology of these disorders as well as the dystonic spread remain largely unknown.

Increased neuroimaging studies have shown that dystonia results from a dysfunctional network not limited to the traditional view of the basal ganglia (7). Resting-state fMRI measures spontaneous brain functional activity through changes in blood oxygen level dependent signals, commonly involving the metrics of static amplitude of low-frequency fluctuations (sALFF) and static functional connectivity (sFC) (8). BSP patients showed abnormal functional connectivity (FC) in the sensorimotor cortex, frontoparietal network, default mode network, and salience network (9). Furthermore, altered segregation and integration of the cortical-basal ganglia functional network was detected in BSP patients, which might be associated with disease severity (10).

However, static parameters may ignore of the temporal variability in brain activity and connectivity. Dynamic analysis, which combines amplitude of low-frequency fluctuations (ALFF) and FC measurement with the sliding window method, can effectively capture the dynamic characteristics of brain function (11). Dynamic ALFF (dALFF) reflects temporal changes in spontaneous brain functional activity, whereas dynamic functional connectivity (dFC) strength and variability indicate the time-varying average strength and variability of neural signal correlation between two brain regions (12, 13). A recent study revealed that dALFF and dFC were complementary to the static parameters for detecting functional abnormalities in BSP patients, which may involve disruption of the cortico-ponto-cerebello-thalamo-cortical circuit (14). Subsequent lesion coactivation network mapping revealed that the bilateral supplementary motor area (SMA) played an important role in the pathology of BSP (15). However, brain functional changes in BOM have been scarcely explored and a lack of comparative studies between BSP and BOM. Shared abnormal changes would help elucidate the initiation mechanisms in these two disorders, while differences between groups probably reveal the pathophysiology of dystonic spread.

In this study, we combined dALFF and dFC with sALFF and sFC to compare brain activity and connectivity differences among BSP patients, BOM patients and healthy controls (HC) for the first time. We aimed to identify (i) shared functional changes in BSP and BOM patients; (ii) specific changes in each patient group and differences between BSP and BOM; (iii) correlations between dynamic parameters and clinical severity scores. Potential neuroimaging biomarkers might be found to distinguish dystonia patients from HC and BSP from BOM patients, further helping the determination of disease prognosis.

## Materials and methods

### Participants

Sixteen BSP patients and 16 BOM patients were recruited from the Movement Disorders Clinic at Peking Union Medical College Hospital between October 2022 and June 2023. The inclusion criteria were as follows: (1) clinically diagnosed with isolated BSP or BOM according to the published criteria by an experienced neurologist (X.W.) (16); (2) onset age over 20 years; (3) no botulinum toxin injections within 3 months before imaging; and (4) no history of exposure to medications that affect the dopaminergic system or block dopamine receptors within 3 months before imaging. The exclusion criteria included known causes of dystonia, a history of other neurological diseases, Mini-Mental State Examination score <24, severe systematic diseases, severe psychiatric illness, and any contraindication to MRI. Twenty HC matched for age, sex, and education were also enrolled.

This study was approved by the Research Ethics Committee of Peking Union Medical College Hospital (No. I-22PJ481). Written informed consent was obtained from all participants.

### Clinical assessment

Patient demographic and clinical data, including age, sex, education level, disease duration, sensory tricks, disease severity, and duration of botulinum toxin treatment, were collected. The severity of dystonia was assessed using the Burke-Fahn-Marsden Dystonia Rating Scale (BFMDRS) movement score and disability score, the Jankovic Rating Scale (JRS), and the Blepharospasm Disability Index (17–19). The Mini-Mental State Examination was used as a brief cognitive evaluation.

### Image acquisition

All imaging data were acquired on a 3T scanner (Discovery MR750, GE Healthcare, Chicago, IL, USA) with an eight-channel head coil. The participants were instructed to lie still, relax with their eyes closed, and stay awake during the scan. Resting-state fMRI data were obtained using a gradient echo EPI sequence with the following parameters: repetition time (TR) = 2,000 ms, echo time = 30 ms, field of view =240 mm × 240 mm, flip angle = 90°, matrix = 64 mm × 64 mm, and slice thickness = 4 mm. Three-dimensional T1WI was collected with the following parameters: repetition time = 7.5 ms, echo time = 3.2 ms, inversion time = 400 ms, number of excitations = 1, field of view = 256 mm × 256 mm, flip angle = 12°, and matrix = 256 mm × 256 mm. Clinical sequences including T2WI, FLAIR, DWI, T2^*^-weighted images, and MRA, were also performed to exclude structural abnormalities.

### Image preprocessing

Resting-state fMRI images were preprocessed using the Data Processing and Analysis of Brain Imaging toolbox (DPABI v4.3; http://rfmri.org/dpabi) via MATLAB R2019b (MathWorks Inc., Natick, MA, USA). The first 10 volumes were discarded to ensure the equilibrium of the blood oxygen level dependent signal, and the remaining data were corrected for slice timing and head motion. Subjects with translational movement >2 mm or rotational movement >2° were excluded. Then, the blood oxygen level dependent image of each participant was coregistered to its corresponding T1WI, normalized to the Montreal Neurological Institute space according to T1WI, and resampled at a voxel size of 3 × 3 × 3 mm^3^. The image was further spatially smoothed with a 6 mm full width at half maximum Gaussian kernel. Finally, covariates, including Friston-24 head motion parameters, cerebrospinal fluid signals, and white matter signals, were regressed out.

### sALFF and dALFF analysis

The DPABI v4.3 toolbox was used to compute sALFF, dALFF, sFC, and dFC. The sALFF calculation included the following steps: for each voxel, the time series was converted into the frequency domain with a fast Fourier transform, and the power spectrum was acquired; next, the square root of the power spectrum was calculated, and the average square root across 0.01–0.10 Hz at each voxel was taken as the ALFF; finally, the ALFF of each voxel was divided by the global mean ALFF for result standardization, denoted as the sALFF (20).

ALFF was combined with the sliding window method to compute the dALFF, reflecting the temporal variability in brain activity. A sliding window length of 50 TRs and a step size of two TRs were selected to balance the capture of rapid dynamic shifts and the reliability of estimating brain activity (14, 21). The standard deviation of the sALFF across all windows was computed and considered the dALFF map. The dALFF maps were subsequently normalized to z scores to improve the normality of the distribution.

### sFC and dFC analysis

Changes in sFC and dFC were determined with seed-based voxel correlation analyses. Brain regions showing significant intergroup differences in dALFF were selected as seeds for sFC and dFC analysis. To generate sFC maps, the mean time course of each seed was calculated and correlated with all voxels of the entire brain. Consistent with dALFF variance analysis, dFC maps were generated by calculating correlation coefficients between each seed and the rest of the brain in each sliding window. The intensity and temporal variability of dFC fluctuations were reflected, respectively by dFC strength and dFC variability, respectively.

### Statistical analysis

Demographic and clinical data were analyzed using SPSS (version 26.0, IBM Corp, Armonk, NY, USA). Normally distributed continuous variables were expressed as the means ± standard deviations and were compared among the three groups using one-way analysis of variance. Skewed variables were presented as medians and interquartile ranges. Nonparametric Kruskal–Wallis H tests were used for three-group comparisons, and the Mann–Whitney *U*-test was used for two-group comparisons. Categorical data were analyzed with Fisher's exact test. Associations between dynamic parameters (dALFF and dFC) and clinical scores (BFMDRS movement score, JRS and blepharospasm disability index) were analyzed using Spearman partial correlation after adjusting for age, sex, years of education, and duration of disease as covariates. The significance level was set at two-tailed *p* < 0.05.

Differences in neuroimaging parameters (sALFF, dALFF, sFC, dFC strength, and dFC variability) among the three groups were compared using one-way analysis of variance with DPABI v4.3. Disease duration, BFMDRS movement score, and total intracranial volume were included as covariates, and *post-hoc* analysis was conducted to compare groups in pairs. Intergroup comparisons were considered statistically significant at a voxel *p* < 0.001 and cluster *p* < 0.05, with Gaussian random field correction.

### Validation analysis

To verify our findings regarding dFC strength using a sliding window length of 50 TRs, auxiliary analyses of different sliding window lengths (30 and 70 TRs) were also conducted.

## Results

### Demographic and clinical characteristics

The demographic and clinical characteristics of the BSP, BOM and HC groups are summarized in Table 1. No significant differences were found in age, sex, or years of education among the three groups. Compared with the BSP group, the BOM group had a significantly longer disease duration, higher BFMDRS movement and disability scores, and a lower percentage of sensory tricks. However, no significant differences in JRS score, Blepharospasm Disability Index score, or duration of botulinum toxin treatment were detected between the BSP and BOM groups.

**Table 1:** Demographic and clinical characteristics.

**Demographic and clinical information**	**BSP *n* = 16**	**BOM *n* = 16**	**HC *n* = 20**	***p*-Value**
Median age, y (IQR)	53.0 (46.3–60.8)	52.5 (47.3–64.3)	58.0 (52.5–61.0)	0.512
Male, *n* (%)	4 (25)	4 (25)	7 (35)	0.796
Mean education, *y* (SD)	9.5 (5.8)	10.3 (4.8)	10.4 (2.5)	0.796
Median duration, *m* (IQR)	16.0 (7.0–76.3)	47.0 (34.5–114.0)		0.010
Median BFMDRS movement, (IQR)	8.0 (6.1–8.4)	9.0 (7.0–12.0)		0.020
Median BFMDRS disability, (IQR)	0.0 (0.0–0.0)	0.0 (0.0–1.0)		0.007
Median JRS, (IQR)	6.0 (6.0–7.0)	6.0 (5.3–6.0)		0.066
Median BSDI, (IQR)	16.0 (7.3–18.8)	8.0 (5.3–11.5)		0.052
Sensory tricks, *n* (%)	15 (93.8)	4 (25.0)		0.000
Median BoNT duration, *m* (IQR)	0.0 (0.0–13.5)	9.0 (0.0–90.0)		0.156

BSP, blepharospasm; BOM, blepharospasm-oromandibular dystonia; HC, healthy controls; IQR, interquartile range; SD, standard deviation; y, years; n, number; m, months; BFMDRS, Burke-Fahn-Marsden Dystonia Rating Scale; JRS, Jankovic Rating Scale; BSDI, blepharospasm disability index; BoNT, botulinum toxin.

### Comparison of sALFF and dALFF

**Figure 1:**
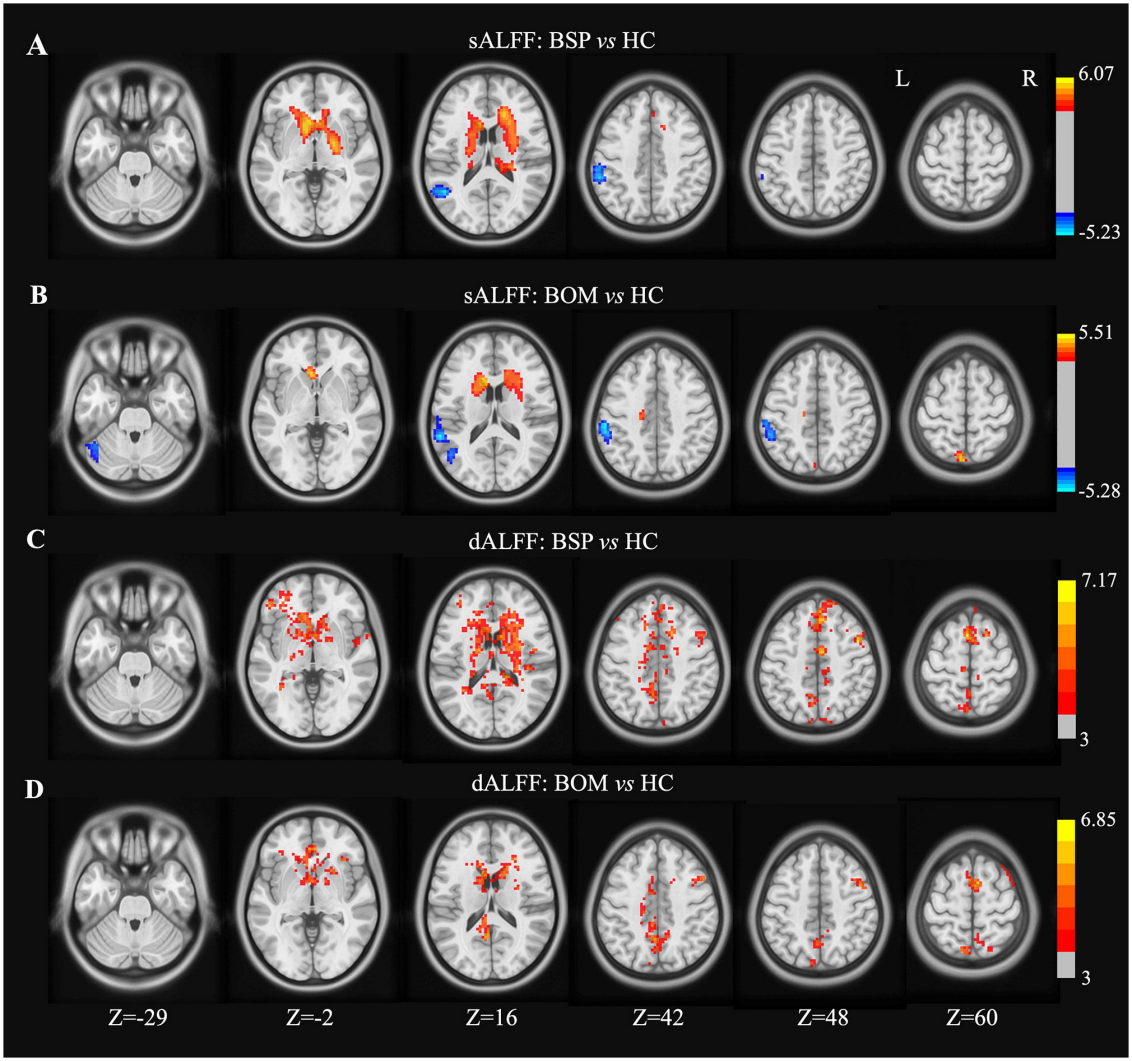
Brain regions showing significant differences in sALFF and dALFF variance between groups. **(A)** sALFF differences between BSP and HC; **(B)** sALFF differences between BOM and HC; **(C)** dALFF differences between BSP and HC; **(D)** dALFF differences between BOM and HC. Color bar indicates range of *t*-values. L, left; R, right.

Compared with HC, both BSP and BOM patients presented significantly increased sALFF in the bilateral caudate, and decreased sALFF in the left middle temporal gyrus and inferior parietal lobe (Figures 1A, B and Supplementary Table S1). The BSP patients also showed increased sALFF in the bilateral putamen and right pallidum (Figure 1A and Supplementary Table S1). In contrast, increased sALFF in the left precuneus and decreased sALFF in the left cerebellar crus I were found in the BOM group (Figure 1B and Supplementary Table S1). In addition to overlapping regions with increased sALFF, both BSP and BOM patients displayed increased dALFF in the bilateral SMA, right precentral gyrus, and bilateral cingulate gyrus compared with HC (Figures 1C, D and Supplementary Table S1). Increased dALFF was observed in the left middle frontal gyrus in BSP patients and in the right superior frontal gyrus in BOM patients (Figures 1C, D and Supplementary Table S1). Direct comparison of BSP and BOM patients did not reveal significant differences in sALFF and dALFF.

### Comparison of sFC and dFC

The right precentral gyrus, left SMA, and right pallidum were used as seeds for sFC and dFC analysis. In these three seeds, there were no significant differences in sFC or dFC strength between the BSP and HC groups. There were also no significant differences in dFC variability among the BSP, BOM and HC groups.

**Figure 2:**
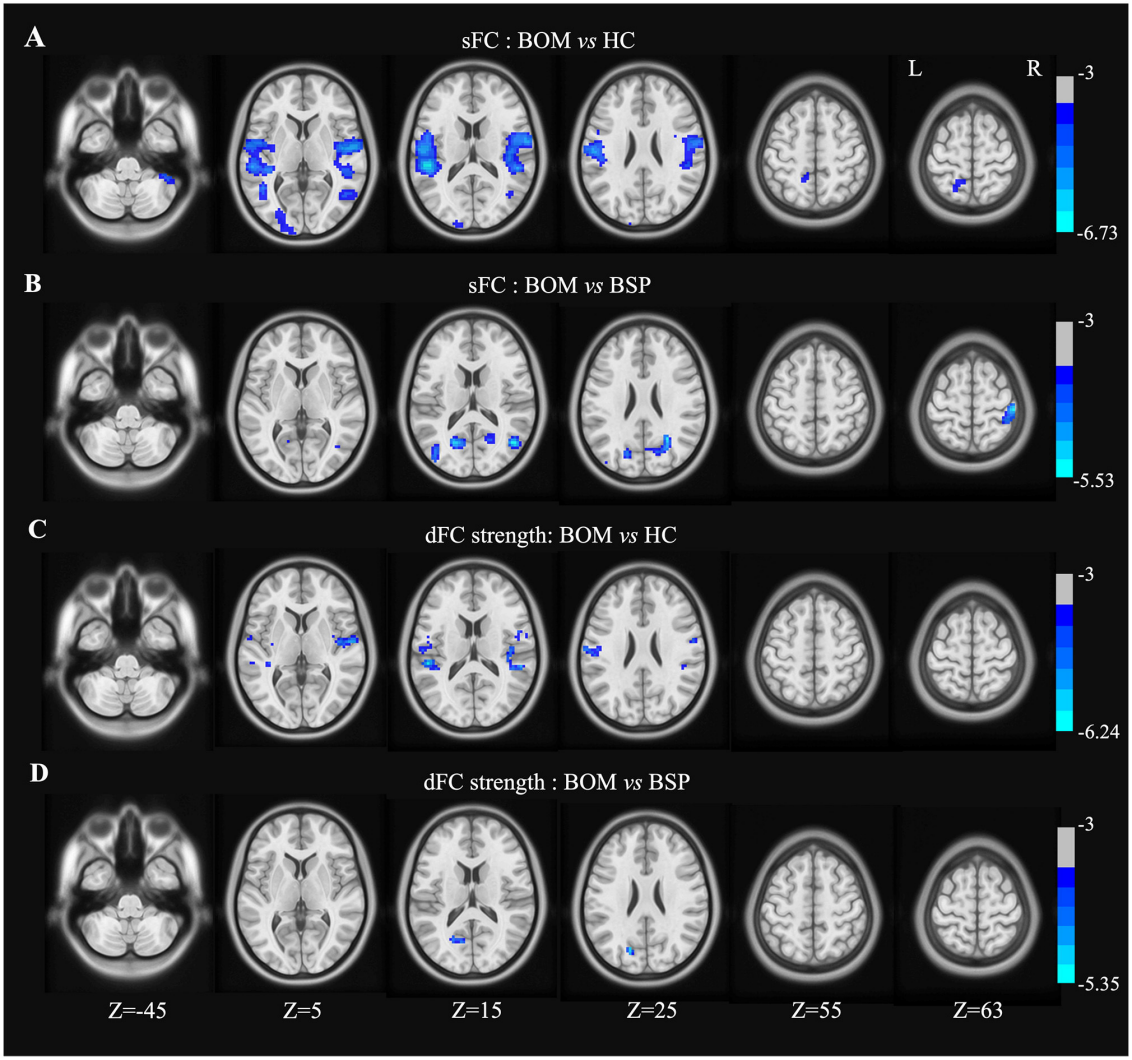
Brain regions showing significant differences in sFC and dFC strength with right precentral gyrus between groups. **(A)** Decreased sFC between BOM and HC; **(B)** Decreased sFC between BOM and BSP; **(C)** Decreased dFC strength between BOM and HC; **(D)** Decreased dFC strength between BOM and BSP. Color bar indicates range of *t*-values. L, left; R, right.

In the right precentral gyrus seed, BOM patients presented decreased sFC in the bilateral postcentral gyrus, rolandic operculum, temporal lobe, left parietooccipital lobe, and right cerebellar crus II compared with HC (Figure 2A and Supplementary Table S2). Furthermore, compared with BSP patients, BOM patients exhibited decreased sFC in the right postcentral gyrus, right temporal lobe, and bilateral occipital lobe (Figure 2B and Supplementary Table S2). The brain regions with decreased dFC strength had a similar but limited pattern in BOM vs. HC and BOM vs. BSP comparisons (Figures 2C, D and Supplementary Table S2).

**Figure 3:**
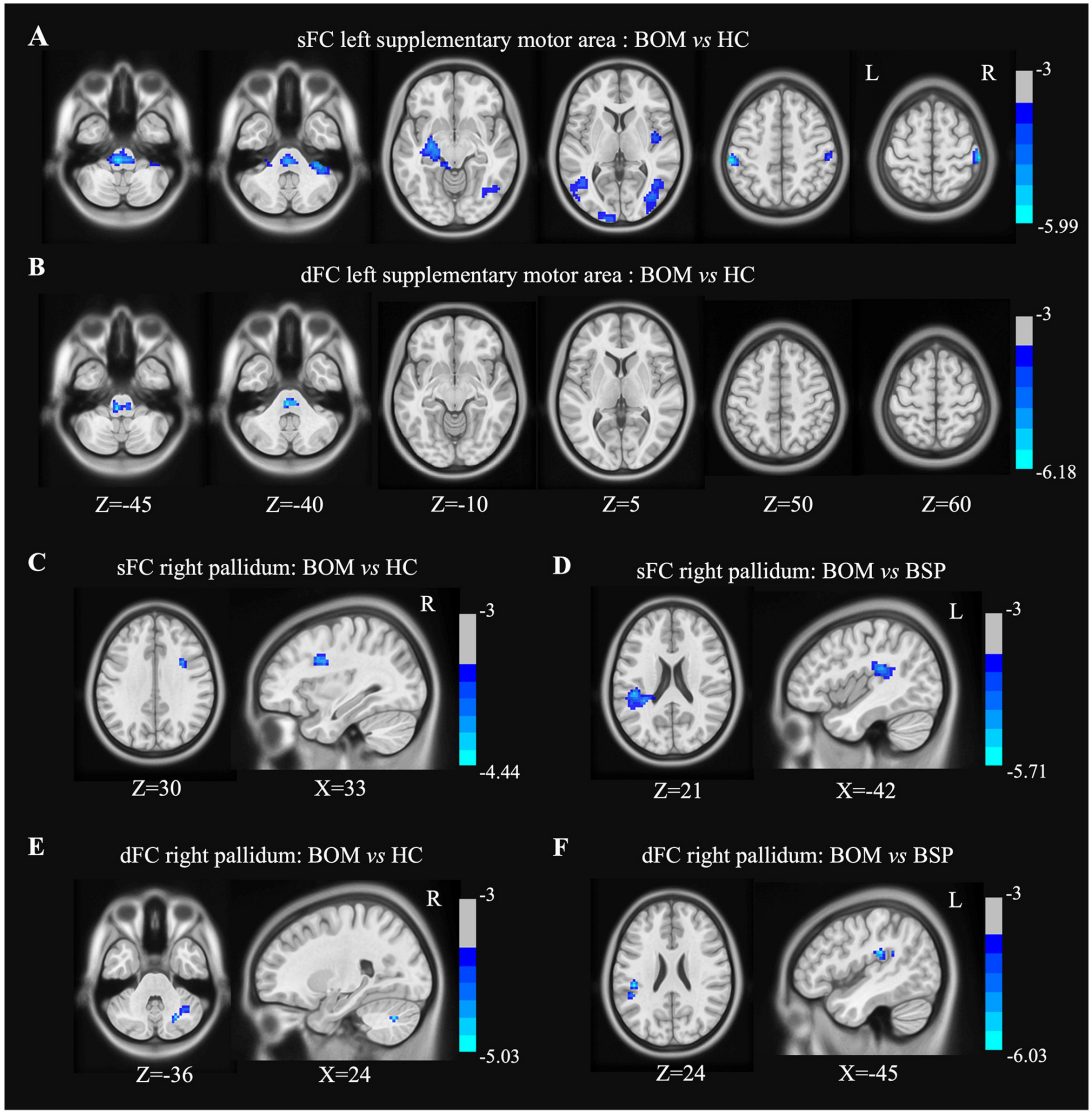
Brain regions showing significant differences in sFC and dFC strength with left SMA and right pallidum between groups. **(A)** Decreased sFC with left SMA between BOM and HC; **(B)** Decreased dFC strength with left SMA between BOM and HC; **(C)** Decreased sFC with right pallidum between BOM and HC. **(D)** Decreased sFC with right pallidum between BOM and BSP; **(E)** Decreased dFC with right pallidum between BOM and HC. **(F)** Decreased dFC with right pallidum between BOM and BSP. Color bar indicates range of *t*-values. L, left; R, right.

In the left SMA seed, BOM patients exhibited decreased sFC in the brainstem, right cerebellum, right postcentral gyrus, bilateral temporal lobe, right insula and left parietooccipital lobe compared with HC (Figure 3A and Supplementary Table S3). Compared with HC, BOM patients exhibited decreased dFC strength only in the brainstem (Figure 3B and Supplementary Table S3). No significant differences in sFC or dFC strength were detected between BSP and BOM patients.

In the right pallidum seed, BOM patients exhibited decreased sFC in the opercular part of the right inferior frontal gyrus compared to HC and in the left inferior parietal lobule compared to BSP patients (Figures 3C, D and Supplementary Table S4). Furthermore, BOM patients exhibited decreased dFC strength in the right cerebellar crus I compared to HC and in the left supramarginal gyrus compared to BSP patients (Figures 3E, F and Supplementary Table S4).

### Correlations between altered dALFF and dFC and clinical parameters

A significant negative correlation between BFMDRS movement scores and dALFF in the left middle cingulate gyrus was detected in BSP patients. JRS scores were negatively correlated with dALFF in the right precentral gyrus, bilateral SMA, left precuneus, and bilateral middle cingulate gyri in BSP patients. In contrast, JRS scores and dALFF in the right putamen were negatively correlated in BOM patients (Table 2). No significant correlations between either clinical scores and dFC strength or blepharospasm disability index scores and dynamic parameters were found.

**Table 2:** Correlations between dynamic parameters and clinical scores in BSP and BOM groups.

**Groups**	**Dynamic parameters and clinical scores**	**Regions**	***r***	***p*-Value**
BSP	dALFF and BFMDRS movement	Left middle cingulate gyrus	−0.637	0.026
	dALFF and JRS	Right precentral gyrus	−0.679	0.015
		Left supplementary motor area	−0.596	0.041
		Right supplementary motor area	−0.679	0.012
		Left precuneus	−0.648	0.023
		Left middle cingulate gyrus	−0.657	0.020
		Right middle cingulate gyrus	−0.606	0.037
BOM	dALFF and JRS	Right putamen	−0.749	0.005

BSP, blepharospasm; BOM, blepharospasm-oromandibular dystonia; dALFF, dynamic amplitude of low-frequency fluctuations; BFMDRS, Burke-Fahn-Marsden Dystonia Rating Scale; JRS, Jankovic Rating Scale.

### Validation results

The results for sliding window lengths of 30 and 70 TRs confirmed our main findings of dFC strength (Supplementary Tables S5, S6).

## Discussion

To our knowledge, this is the first study comparing the functional brain changes between the BSP and BOM group by combining the static and dynamic resting-state fMRI metrics. Compared with HC, both BSP and BOM patients presented increased dALFF in the bilateral basal ganglia, bilateral SMA, right precentral gyrus, and bilateral cingulate gyrus. BOM further demonstrated decreased sALFF in the left cerebellum. In the FC analysis, BOM is featured with functional abnormality from these key regions to widespread connectivity reduction in the sensorimotor cortico-basal ganglia-brainstem-cerebellar network. Clinical scores of BSP severity were significantly correlated with dALFF in some of these important regions.

In this study, we found increased sALFF and dALFF in the bilateral basal ganglia in BSP and BOM patients. Increased dALFF represents excessive variability in brain activity, which may trigger specific pathological states, and indicates impaired brain function (22, 23). Thus, increased sALFF and dALFF values suggested that BSP and BOM might exhibit not only increased neural activity but also brain function disorders in the basal ganglia.

We also observed increased dALFF in the right precentral gyrus in BSP and BOM patients. This finding in BSP patients is consistent with a previous study (14), while firstly identified in BOM patients. These results suggest a shared brain impairment pattern affecting the stability of the right primary motor cortex. Dysfunction of the primary motor cortex may reduce the peripheral inhibition, causing excessive muscle contractions and involuntary movements (24). Thus, impaired brain function in the right precentral gyrus supports early neuroelectrophysiology findings in BSP and BOM patients (5).

This study also revealed increased dALFF in the bilateral SMA and middle cingulate gyrus in both patient groups. The bilateral SMA is involved in visual motion, imagination, action execution, finger tapping, and eye movements (15). Lesion coactivation network mapping revealed the main involvement of the bilateral SMA in lesion-induced BSP, and these areas exhibited reduced FC with the left posterior cingulate cortex in idiopathic BSP (15). Structural covariance networks and previous fMRI studies also supported the pathological changes in the SMA in BSP patients (8, 25, 26). Our findings further highlight the critical role of the bilateral SMA in BOM patients. In BSP patients, various fMRI studies have revealed abnormal FC of the posterior or anterior cingulate gyrus with other brain regions (10, 27, 28). A randomized controlled study showed transcranial magnetic stimulation targeting anterior cingulate cortex could improve clinical symptoms of BSP patients immediately but briefly (29). This study suggested impaired brain activity in the middle cingulate gyrus in both BSP and BOM patients. The middle cingulate gyrus is involved in emotion, movement, and memory functions (30). It integrates the reward value outcome information from the anterior cingulate gyrus and action information from the posterior cingulate gyrus and transmits the results to premotor cortical areas (30). Further research is needed to explore the middle cingulate gyrus as a potential treatment target in BSP and BOM patients.

In addition to the overlapping brain regions with abnormal activity, the BOM group exhibited decreased sALFF in the left cerebellar crus I, which was not observed in the BSP group. This finding indicates a specific role of cerebellar dysfunction in the pathogenesis of BOM, potentially participating in the progression of dystonia from BSP to the oromandibular regions.

Using the right precentral gyrus, left SMA, and right pallidum as seeds, no significant sFC or dFC changes were detected in BSP patients. In contrast, BOM patients is featured with widespread connectivity reduction. BSP patients have been reported to exhibit decreased dFC between the right precentral gyrus and right cerebellar hemisphere (14). The significance threshold was stricter in our study, possibly contributing to the inconsistent results between the two studies. Analysis of a mixed cohort including 13 patients with BSP or BOM revealed widespread decreases in sFC in the basal ganglia, cerebellum, primary and secondary sensory motor cortices, and visudal-related brain areas (31). Our study further differentiated these two groups and revealed more extensive cortical-brainstem-cerebellar and cortical-basal ganglia-cerebellar FC reductions in BOM patients than in BSP patients when compared with HC. Direct comparison between BOM and BSP patients revealed reduced FC involving the primary motor cortex, somatosensory cortex, globus pallidus, and temporooccipital regions. Our findings highlighted that BOM patients had more widespread and severe abnormalities in functional networks than BSP patients did. Research has revealed that the pathophysiology of dystonia involves dysfunction of the cortico-basal ganglia-thalamo-cortical loop and cortico-ponto-cerebello-thalamo-cortical circuit (32). We further discovered decreased FC between the right basal ganglia and right cerebellar crus I in BOM patients. Reduced cerebellar connectivity can also weaken the inhibitory function of the sensorimotor cortex, causing excessive involuntary movements (33).

In this study, both BSP and BOM displayed abnormal brain activity in the precentral gyrus and SMA. However, only BOM patients showed reduced FC between the precentral gyrus/SMA and postcentral gyri compared with HC. Significant FC reduction was not found between the sensory and motor cortex in BSP. Direct comparison of BOM and BSP patients also revealed reduced FC between the right precentral gyrus and postcentral gyrus, indicating the specific impairment of sensorimotor integration in BOM. The role of impaired sensorimotor integration has gradually been recognized in the pathophysiology of dystonia (26). A prospective study found that the mandibular branch P1-N2 peak interval and amplitude of the trigeminal somatosensory evoked potential served as independent risk factors for spread of BSP (4). These parameters reflect the pathway activity of thalamic-basal ganglia to the primary sensory cortex. Combing with our study, these results further indicate that sensory pathway abnormalities and disruption of the sensorimotor integration might lead to the spread of BSP to the lower face muscles. A BOM patient was successfully treated with navigated repetitive transcranial magnetic stimulation targeting the precentral gyrus (34). In addition to possible symptom relief, this study suggested that therapeutic modulation of sensorimotor cortex by noninvasive stimulation technologies may also prevent dystonic spread.

In a recent structural MRI study, the thalamus was found to be closely associated with the onset of both BSP and BOM (35). Consistent with another fMRI study, we did not find significant sALFF or dALFF changes in the thalamus in BSP or BOM patients (14). Future exploration of thalamic functional changes in BSP and BOM is need, which could focus on thalamus-based FC, metabolism and perfusion changes.

Correlation analysis revealed that in BSP patients, brain activities of abnormal regions were also correlated with disease severity, involving the right precentral gyrus, bilateral SMA and middle cingulate gyrus. In BOM patients, dALFF in the right putamen was significantly associated with the severity of blepharospasm. Our results indicate that functional changes in the motor cortex, basal ganglia, and cingulate gyrus may not only contribute to the disease pathogenesis but also be imaging biomarkers of BSP severity. It has been suggested that cerebello-cortical circuits may be correlated with the severity of BSP, whereas basal ganglia circuits are associated with triggering of eyelid spasms (36). A recent study proposed that the disease severity of BSP patients may be related to cortical-basal ganglia network dysfunction (10). Conversely, another fMRI study revealed no significant correlation between dynamic parameters and disease severity scores in BSP patients (14). Thus, using of imaging parameters in assessing the clinical severity of BSP and BOM still has limitations. Future longitudinal studies involving larger cohorts may help identify imaging biomarkers related to clinical parameters.

Several limitations should be considered in this study. First, the selection of the sliding window length is still controversial, and the optimal window length for capturing brain dynamic activity remains unclear. Based on previous neuroimaging studies, we selected 50 TRs as the standard sliding window length (14, 21). Furthermore, validation analyses of 30- and 70-TR sliding window lengths yielded similar results, indicating the relative stability of our dynamic results. Second, the sample size was relatively small and might have affected the power of the intergroup comparisons; larger cohort studies are needed to confirm our findings. Finally, we included only HC as the control group. Comparing BSP and BOM patients with hemifacial spasm patients may help identify brain functional changes due to excessive facial movement. Nonetheless, this study directly compared BSP and BOM patients, partially eliminating the influence of excessive facial movement on the results.

## Conclusions

This study combined dynamic and static resting-state fMRI metrics to provide more comprehensive insights into the pathogenesis of BSP and BOM. Our results demonstrated that impaired functional activity in the right precentral gyrus, bilateral SMA, basal ganglia, and cingulate gyrus is the common pathophysiological basis for BSP and BOM patients. Dynamic ALFF in these key regions might also be potential imaging biomarkers of BSP severity. Further, BOM is featured with functional abnormality from these key regions to widespread connectivity reduction in the sensorimotor cortico-basal ganglia-brainstem-cerebellar network. Our findings highlighted the common pathophysiological mechanisms in BSP and BOM and preliminarily revealed the unique functional changes in BOM. These knowledges could help investigate mechanisms of dystonia spread and potentially facilitate disease-modifying therapies.

## Data Availability

The original contributions presented in the study are included in the article/Supplementary material, further inquiries can be directed to the corresponding author.
