# Supplementary material for: Comparative study of brain activity and functional connectivity in blepharospasm and blepharospasm-oromandibular dystonia
Source: Front Neurol. 2025 Jul 3;16:1583297. doi: 10.3389/fneur.2025.1583297 (PMC12268265; doi:10.3389/fneur.2025.1583297)
Supplement: Supplementary file 1 [file Table_1.doc]

**Supplementary Table 1 Brain regions showing significant differences in sALFF and dALFF variance between groups**

| Groups | Regions | Side | Cluster size | Peak MNI coordinate | | | T |
| --- | --- | --- | --- | --- | --- | --- | --- |
|  |  |  |  | X | Y | Z |  |
| Differences in sALFF |  |  |  |  |  |  |  |
| BSP＞HC | Caudate  Putamen  Pallidum | B  B  R | 2066 | -12 | 12 | 3 | 6.0734 |
| BSP＜HC | Middle temporal gyrus  Inferior parietal lobe | L  L | 467 | -48 | -57 | 15 | -5.2292 |
| BOM＞HC | Caudate | L | 494 | -9 | -12 | 27 | 5.5146 |
|  | Caudate | R | 374 | 21 | 3 | 24 | 5.1644 |
|  | Precuneus | L | 63 | -3 | -75 | 57 | 5.5141 |
| BOM＜HC | Inferior parietal lobe  Middle temporal gyrus | L  L | 565 | -57 | -30 | 45 | -5.2844 |
|  | Cerebellar crus I | L | 61 | -45 | -54 | -27 | -4.7790 |
| Differences in dALFF |  |  |  |  |  |  |  |
| BSP＞HC | Precuneus  Caudate  Supplementary motor area  Putamen  Middle cingulate gyrus  Pallidum | L  B  B  B  B  R | 4617 | 15 | 6 | 3 | 7.1739 |
|  | Precentral gyrus | R | 109 | 45 | 6 | 48 | 6.8105 |
|  | Middle frontal gyrus | L | 115 | -51 | 30 | 30 | 5.6330 |
| BOM＞HC | Precuneus  Caudate  Middle cingulate gyrus | L  B  L | 1847 | -3 | -36 | 39 | 6.8526 |
|  | Supplementary motor area | B | 125 | 6 | 6 | 60 | 5.8252 |
|  | Precentral gyrus | R | 98 | 45 | 6 | 48 | 5.4050 |
|  | Superior frontal gyrus, orbital part | R | 60 | 21 | 36 | -24 | 4.8358 |

Abbreviations: BSP, blepharospasm; BOM, blepharospasm-oromandibular dystonia; HC, healthy controls; sALFF, static amplitude of low-frequency fluctuations; dALFF, dynamic amplitude of low-frequency fluctuations; L, left; R, right; B, bilateral; MNI, Montreal Neurological Institute standard space.

**Supplementary Table 2 Brain regions showing significant differences in sFC and dFC strength with right precentral gyrus between groups**

| Groups | Regions | Side | Cluster size | Peak MNI coordinate | | | T |
| --- | --- | --- | --- | --- | --- | --- | --- |
|  |  |  |  | X | Y | Z |  |
| Differences in sFC |  |  |  |  |  |  |  |
| BOM＜HC | Cerebellar crus II | R | 52 | 45 | -42 | -45 | -4.6636 |
|  | Rolandic operculum  Superior temporal gyrus  Postcentral gyrus | R  R  R | 1082 | 54 | -9 | 9 | -5.6321 |
|  | Superior temporal gyrus  Postcentral gyrus  Middle temporal gyrus  Rolandic operculum | L  L  L  L | 1624 | -51 | -33 | 15 | -6.7284 |
|  | Middle occipital gyrus | L | 260 | -27 | -78 | 3 | -4.7480 |
|  | Middle temporal gyrus | R | 105 | 45 | -63 | 9 | -4.4176 |
|  | Precuneus | L | 108 | -12 | -48 | 57 | -4.2965 |
| BOM＜BSP | Middle temporal gyrus | R | 64 | 45 | -66 | 15 | -5.4657 |
|  | Calcarine fissure and surrounding cortex | L | 109 | -21 | -66 | 15 | -5.1993 |
|  | Cuneus | R | 146 | 27 | -60 | 27 | -5.5314 |
|  | Middle occipital gyrus | L | 55 | -42 | -78 | 18 | -4.5649 |
|  | Postcentral gyrus | R | 129 | 48 | -27 | 63 | -5.3238 |
| Differences in dFC |  |  |  |  |  |  |  |
| BOM＜HC | Rolandic operculum  Superior temporal gyrus | R  R | 202 | 54 | -36 | 18 | -5.3607 |
|  | Superior temporal gyrus  Postcentral gyrus | L  L | 362 | -51 | -33 | 15 | -6.2364 |
| BOM＜BSP | Superior occipital gyrus | L | 57 | -18 | -75 | 24 | -5.3520 |

Abbreviations: BSP, blepharospasm; BOM, blepharospasm-oromandibular dystonia; HC, healthy controls; sFC, static functional connectivity; dFC, dynamic functional connectivity; L, left; R, right; MNI, Montreal Neurological Institute standard space.

**Supplementary Table 3 Brain regions showing significant differences in sFC and dFC strength with left supplementary motor area between groups**

| Groups | Regions | Side | Cluster size | Peak MNI coordinate | | | T |
| --- | --- | --- | --- | --- | --- | --- | --- |
|  |  |  |  | X | Y | Z |  |
| Differences in sFC |  |  |  |  |  |  |  |
| BOM＜HC | Pons, Medulla | B | 215 | -6 | -33 | -45 | -5.8164 |
|  | Cerebellar VI | R | 90 | 33 | -36 | -39 | -5.1563 |
|  | Hippocampus | L | 243 | -27 | -12 | -9 | -5.1857 |
|  | Middle temporal gyrus | R | 370 | 45 | -60 | 0 | -4.668 |
|  | Middle temporal gyrus | L | 114 | -42 | -60 | 6 | -4.345 |
|  | Insula | R | 155 | 42 | -6 | 3 | -4.4918 |
|  | Middle occipital gyrus | L | 80 | -9 | -105 | 9 | -4.2404 |
|  | Inferior parietal lobe | L | 99 | -60 | -30 | 51 | -5.9918 |
|  | Postcentral gyrus | R | 58 | 54 | -27 | 60 | -5.7686 |
| Differences in dFC |  |  |  |  |  |  |  |
| BOM＜HC | Pons, Medulla |  | 103 | -6 | -36 | -48 | -6.1801 |

Abbreviations: BSP, blepharospasm; BOM, blepharospasm-oromandibular dystonia; HC, healthy controls; sFC, static functional connectivity; dFC, dynamic functional connectivity; L, left; R, right; MNI, Montreal Neurological Institute standard space.

**Supplementary Table 4 Brain regions showing significant differences in sFC and dFC strength with right pallidum between groups**

| Groups | Regions | Side | Cluster size | Peak MNI coordinate | | | T |
| --- | --- | --- | --- | --- | --- | --- | --- |
|  |  |  |  | X | Y | Z |  |
| Differences in sFC |  |  |  |  |  |  |  |
| BOM＜HC | Inferior frontal gyrus, opercular part | R | 50 | 33 | 9 | 30 | -4.4414 |
| BOM＜BSP | Inferior parietal lobule | L | 174 | -33 | -36 | 27 | -5.7095 |
| Differences in dFC |  |  |  |  |  |  |  |
| BOM＜HC | Cerebellar crus I | R | 32 | 24 | -66 | -36 | -5.0347 |
| BOM＜BSP | Supramarginal gyrus | L | 46 | -45 | -30 | 24 | -6.0266 |

Abbreviations: BSP, blepharospasm; BOM, blepharospasm-oromandibular dystonia; HC, healthy controls; sFC, static functional connectivity; dFC, dynamic functional connectivity; L, left; R, right; MNI, Montreal Neurological Institute standard space.

**Supplementary Table 5 Brain regions showing significant differences in dFC strength with the sliding window length of 30 TRs between groups**

| Groups | Regions | Side | Cluster size | Peak MNI coordinate | | | T |
| --- | --- | --- | --- | --- | --- | --- | --- |
|  |  |  |  | X | Y | Z |  |
| Differences in dFC with right precentral gyrus | |  |  |  |  |  |  |
| BOM＜HC | Superior temporal gyrus  Rolandic operculum | R  R | 168 | 54 | -36 | 18 | -5.5462 |
|  | Superior temporal gyrus  Postcentral gyrus | L  L | 149 | -54 | -9 | -3 | -5.6667 |
| BOM＜BSP | Superior occipital gyrus | L | 45 | -18 | -75 | 24 | -5.399 |
| Differences in dFC with left supplementary motor area | |  |  |  |  |  |  |
| BOM＜HC | Pons, Medulla |  | 112 | -6 | -36 | -48 | -6.1424 |
| Differences in dFC with right pallidum | |  |  |  |  |  |  |
| BOM＜HC | Cerebellar crus I | R | 39 | 24 | -66 | -36 | -4.98 |
| BOM＜BSP | Supramarginal gyrus | L | 45 | -45 | -30 | 24 | -5.4006 |

Abbreviations: TR, repetition time; BSP, blepharospasm; BOM, blepharospasm-oromandibular dystonia; HC, healthy controls; dFC, dynamic functional connectivity; L, left; R, right; MNI, Montreal Neurological Institute standard space.

**Supplementary Table 6 Brain regions showing significant differences in dFC strength with the sliding window length of 70 TRs between groups**

| Groups | Regions | Side | Cluster size | Peak MNI coordinate | | | T |
| --- | --- | --- | --- | --- | --- | --- | --- |
|  |  |  |  | X | Y | Z |  |
| Differences in dFC with right precentral gyrus | |  |  |  |  |  |  |
| BOM＜HC | Rolandic operculum  Superior temporal gyrus | R  R | 230 | 45 | -6 | 3 | -5.2102 |
|  | Superior temporal gyrus | L | 171 | -51 | -33 | 15 | -6.355 |
|  | Postcentral gyrus | L | 100 | -48 | -9 | 21 | -5.0528 |
| BOM＜BSP | Superior occipital gyrus | L | 52 | -18 | -75 | 24 | -5.1541 |
| Differences in dFC with left supplementary motor area | |  |  |  |  |  |  |
| BOM＜HC | Pons, Medulla |  | 125 | -6 | -36 | -48 | -6.4128 |
| Differences in dFC with right pallidum | |  |  |  |  |  |  |
| BOM＜HC | Cerebellar crus I | R | 17 | 36 | -63 | -27 | -4.2665 |
| BOM＜BSP | Supramarginal gyrus | L | 41 | -45 | -30 | 24 | -6.3303 |

Abbreviations: TR, repetition time; BSP, blepharospasm; BOM, blepharospasm-oromandibular dystonia; HC, healthy controls; dFC, dynamic functional connectivity; L, left; R, right; MNI, Montreal Neurological Institute standard space.
